# Postdural Puncture Headaches in Pediatric Patients: A Review of Options When Repeated Epidural Blood Patches Do Not Work

**DOI:** 10.7759/cureus.62833

**Published:** 2024-06-21

**Authors:** Vipin Bansal, Genevieve D'Souza, Emmanuel Aladade, Danielle T McFarlane, Rita Agarwal

**Affiliations:** 1 Anesthesiology, Emory University School of Medicine, Atlanta, USA; 2 Anesthesiology, Stanford Health Care, Stanford, USA; 3 Internal Medicine, Morehouse School of Medicine, Atlanta, USA; 4 Anesthesiology, Stanford University, Stanford, USA

**Keywords:** pediatric-anesthesia, sphenopalatine ganglion block, pain management, epidural blood patch, postdural puncture headache

## Abstract

We present the case of an adolescent with refractory postdural puncture headache (PDPH), whose symptoms resolved with a sphenopalatine ganglion (SPG) nerve block using a J-tip style catheter. Our patient was treated with multiple modalities, including conservative and medical management, multiple epidural blood patches, and different nerve blocks. We discussed different treatments for the PDPH, why each modality did not work, and why our SPG block with a J-tip catheter possibly provided a better sympathetic block in a patient with intractable PDPH for two weeks.

## Introduction

Neuraxial anesthesia has become an essential component in the perioperative management of pediatric surgical pain because it is safe and effective for a variety of ages and situations. The advantages include decreased postoperative nausea, opioid consumption, a stress response to surgery, and superior analgesia. However, serious complications [1] can occur, including postdural puncture headache (PDPH).

PDPH usually manifests within hours up to two weeks following an intentional or unintentional lumbar puncture. Symptoms include a severe headache (often fronto-occipital) that is worse when the patient is upright and improves when lying flat. Other symptoms include nausea, vomiting, hearing changes, photophobia, diplopia, back pain, and neck stiffness.

Treatment of PDPH in adults usually involves initial medical management, followed by an early epidural blood patch (EBP) if medical management fails. Recently, a multi-society international PDPH working group updated its evidence-based clinical practice guidelines for both medical management and interventional options [2]. They found that the success of EBP was overestimated, and the success of EBP in adults is actually between 33% and 91% [2].

Unfortunately, the new adult PDPH guidelines do not provide recommendations for pediatric PDPH. The incidence of PDPH in pediatric patients [3] has been estimated at 1-15% depending on patient factors such as age >10, female gender, increased BMI, and the size of the needle. However, the incidence cited in children is probably low because of the inability of young children to express their symptoms in an objective manner [4].

Like adults, EBP is traditionally considered in pediatric cases where the PDPH fails conservative medical management. In contrast to adults, an EBP in children usually necessitates sedation or general anesthesia because children generally have a fear of needles. However, an EBP does not guarantee the resolution of the PDPH, and the updated success rate of an EBP in pediatrics may be as low as 72% [5]. To improve our understanding of PDPH and its treatment in children, we present a case report of a refractory PDPH that was initially treated with conservative and medical management and multiple EBPs that finally resolved with a sphenopalatine ganglion (SPG) nerve block [6,7] using a J-tip style catheter instead of the traditional cotton swab.

We will discuss the rationale behind the different treatments using the new adult PDPH guidelines as a reference and why our modified SPG block might have improved the PDPH when other traditional medical and procedural interventions failed. Written informed consent, as well as written Health Insurance Portability and Accountability Act (HIPAA) authorization, has been obtained from the patient and guardian for the publication of this case report.

## Case presentation

**Figure 1:**
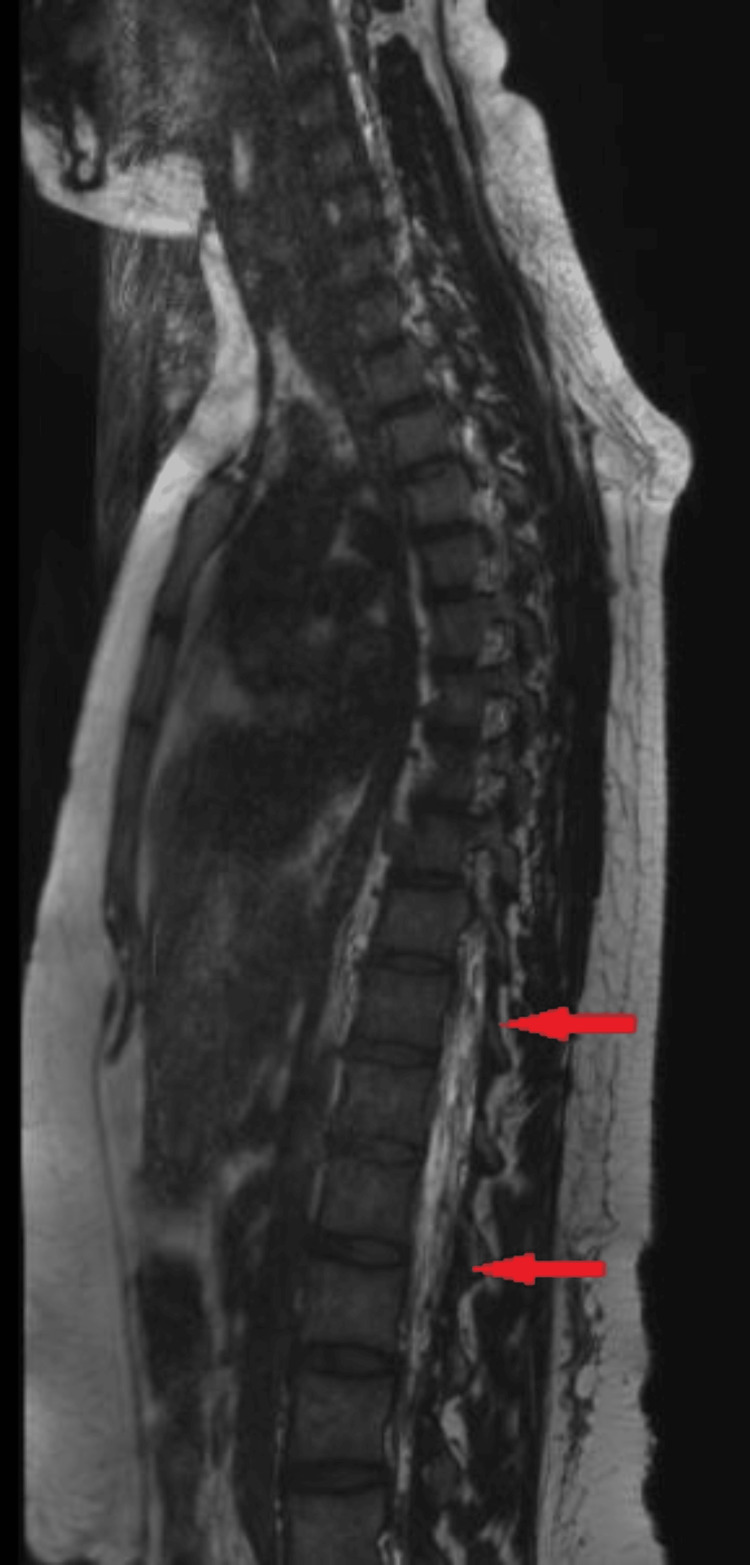
MRI results: diffuse extradural CSF accumulation (within the two arrows) within the thoracolumbar regions with evidence of intracranial hypotension

**Figure 2:**
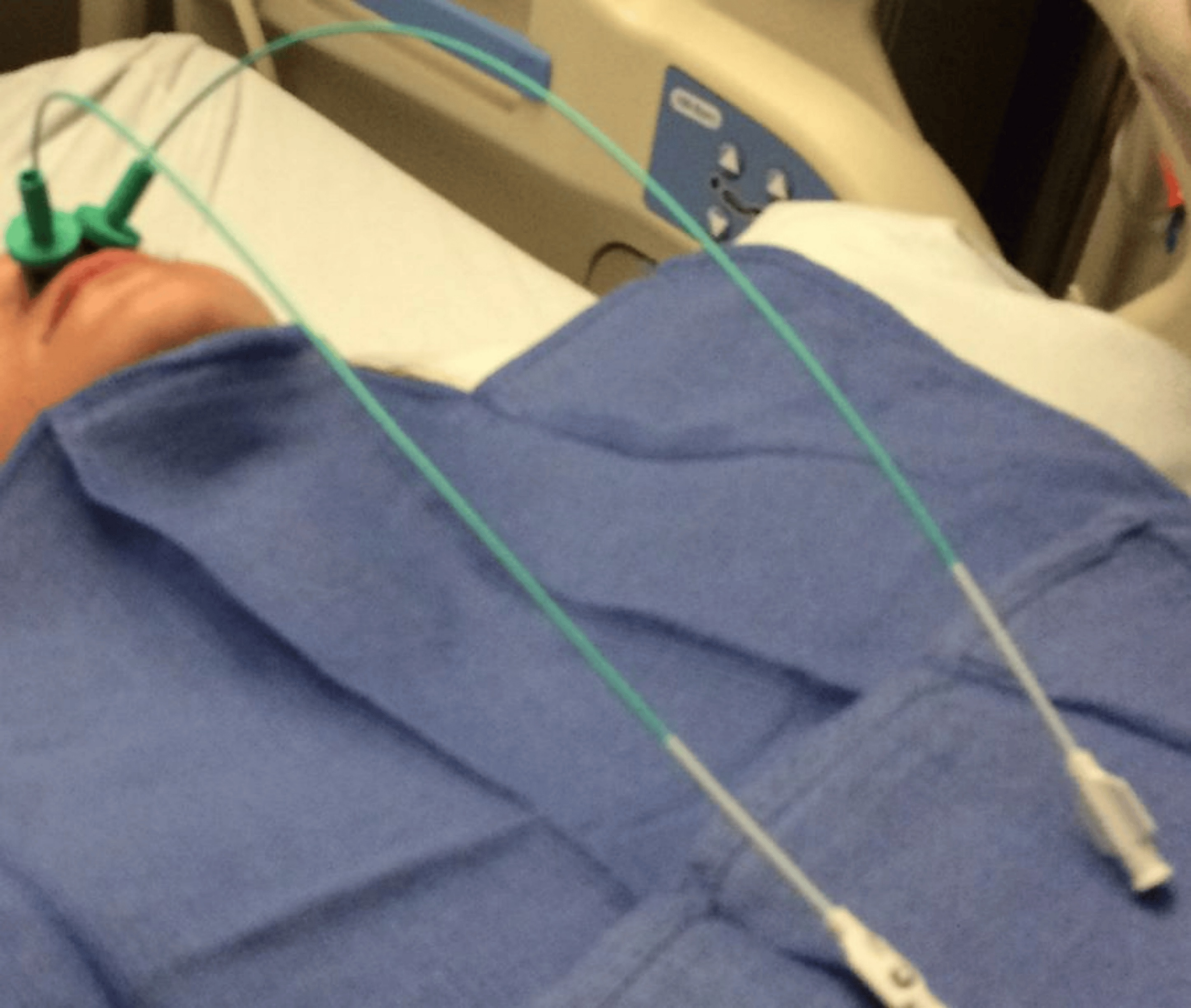
Sphenopalatine block with a J-tip catheter placed through a nasal trumpet

A 14-year-old female patient, 165 cm tall and weighing 68 kg, with localized osteosarcoma, was scheduled for a wide resection of her left tibia and total knee reconstruction. An epidural was placed under general anesthesia for postoperative pain management (Table 1). The epidural placement was difficult, requiring multiple attempts by experienced anesthesia providers. The epidural was initially attempted midline at T12-L1, then at L1-L2, and the final attempt was successful at L2-L3. The epidural was utilized overnight and on the first postoperative day (POD) but was stopped on POD 2 due to the patient’s complaints of positional headaches, nausea, and vomiting. Conservative measures, including bed rest, fluids, caffeine, and cosyntropin, were initially tried. With no resolution of symptoms, MRI imaging was ordered, which revealed diffuse extradural CSF accumulation within the thoracolumbar regions and evidence of intracranial hypotension (Figure 1). On POD 3, occipital nerve blocks and sphenopalatine nerve blocks were placed with a cotton swab. There was no improvement in the patient’s headache, so an EBP was attempted on POD 4, but the headache persisted. A lidocaine infusion was started on POD 6 but was quickly stopped because the patient complained of ringing in her ears and a worsening headache. Another EBP was repeated on POD 8, with minimal improvement in her headaches. A CT myelogram was recommended on POD 9 to pinpoint the leak, but neurosurgery did not want the risk of another dural puncture. On POD 10, the patient could not sit up beyond 10 degrees, so we repeated the SPG block with a J-tip catheter. We employed nasal trumpets to guide the location of the catheter (Figure 2). We improvised our own J-tip using a Glidecath© 4Fr Angle Taper (Terumo, Somerset, United States) from interventional radiology as an alternative to the more costly commercial Tx360® (Tian Medical, Grayslake, United States) [7].

**Table 1:** Ongoing treatment modalities with corresponding outcomes EBP, epidural blood patch; GON, greater occipital nerve; HA, headache; POD, postoperative day; SPG, sphenopalatine ganglion

Day	Events	Comments in the chart
Day of surgery	Wide resection of tibial osteosarcoma	Multiple epidural attempts; final placement OK; epidural started in the recovery area
POD 1	Epidural infusing	Pain controlled
POD 2	Epidural stopped, caffeine started	HA when sitting up, +nausea/vomiting
POD 3	Blocks: (1) bilateral GON and (2) SPG with cotton swabs	No improvement
POD 4	EBP #1	No improvement
POD 5	Bed rest	No change
POD 6	Cosyntropin	No improvement
POD7	Lidocaine infusion	Stopped due to double vision and ringing in the ears
POD 8	EBP #2	Minimal improvement; still has HA when sitting up; still has issues with nausea and vomiting
POD 9	Recommended diagnostic CT myelogram fibrin glue	Declined by neurosurgery (wanted to avoid additional dural punctures)
POD 10	SPG with a catheter	Cannot sit up >10 degrees or eat prior to the procedure. After the procedure, the patient’s HA improved (pain score = 1) and was able to sit up. Nausea and vomiting improved. Tolerated food for the first time in 10 days
POD 11	Follow-up	Patient able to walk to the bathroom
POD 12	Follow up	Patient able to walk around the hallway

The patient was positioned in the supine position, with the head extended. Two nasal trumpets, whose length approximated the length of the nasal passage, were dipped in 2% viscous lidocaine for comfort and placed slowly into each nare. We then advanced a 22G J-tip catheter in each nare, injected 1.5 cc 0.5% ropivacaine bilaterally, and repeated the injections after five minutes with 1 cc 0.5% ropivacaine on each side. A total of 5 cc of 0.5% ropivacaine was used for the procedure. Throughout the procedure, the patient denied any adverse effects, such as ringing in the ears, double vision, nausea, vomiting, or worsening of her headache.

Her PDPH improved significantly, and she was able to sit up in bed. By the following day, she was walking around her room on POD 11. The patient remained symptom free and was discharged a few days later. She was symptom free at two weeks and had a one-month follow-up.

## Discussion

We present the case of a 14-year-old female undergoing lower extremity orthopedic surgery in whom an epidural placement resulted in a severe PDPH that was difficult to manage. We reviewed the pathophysiology of PDPH and the rationale for various common treatment options, and we explored why our modified SPG block succeeded when others were unsuccessful.

The pathophysiology of PDPH is associated with CSF leaking at the puncture site, leading to reduced intracranial volume. This volume reduction appears to trigger the brain to compensate through cerebral vasodilatation. The swelling of the cerebral veins stretches the adjacent pain-sensitive postganglionic parasympathetic fibers, which are believed to trigger the excruciating headache characteristic of PDPH.

Fortunately, most PDPHs improve with conservative management, such as bed rest and fluids for a few days. Medical management is added when the headache does not improve. The main concept behind medical management is to augment CSF production by increasing hydration and using medications such as caffeine [8]. Cosyntropin is a synthetic form of adrenocorticotropic hormone (ACTH) that is believed to stimulate the adrenal gland to increase CSF production [9]. However, the latest international PDPH guidelines [2] do not recommend cosyntropin (ACTH) or other medications such as hydrocortisone, theophylline, sumatriptan, gabapentin, or pregabalin due to a lack of high-quality evidence showing efficacy.

EBP is presumed to seal the leak at the puncture site and has been considered the gold standard [10] for treating refractory PDPH in adults after failed medical management. Our team attempted two EBPs, which did not help. Newer studies now show that the actual success of EBP in adults is between 33% and 91% [2]. In pediatric patients, the incidence of sustained success even with a large single-volume blood patch is around 30-70% [5].

Nerve blocks such as the greater occipital nerve (GON) blocks or SPG blocks have been proposed to treat headaches [11] when EBP is not feasible or does not work. In one study, patients with milder symptoms benefited from the GON block, but those with severe PDPH did not have long-term benefits [12]. The SPG block is thought to work by anesthetizing the SPG, which has synapses adjacent to the postganglionic parasympathetic fibers. The block interrupts the parasympathetic flow in the brain, reducing cerebral vasodilation, which provides relief from PDPH.

The SPG block gained popularity for its simplicity using a cotton swab technique, dipped in viscous lidocaine, and placed in the patient’s nares for several minutes. In obstetric literature [13], treatment of PDPH with SPG block was considered equivalent to EBP. In addition, St. Jude Children’s Research Hospital [14] recommends the SPG block for their patients with PDPH as the initial block when patients have coagulation issues or other contraindications to an EBP.

In the recent adult PDPH guidelines [2], they did not recommend the SPG block. In his 2009 original paper, Cohen commented that the technique was “somewhat unpredictable.” Likewise, our first attempt with the SPG block with a cotton swab was unsuccessful. Several modifications of the SPG method have been proposed to improve the delivery of local anesthetic closer to the SPG, such as hollow-bore cotton swabs [15] and a J-tip catheter [16] to deliver the local anesthetic closer to the SPG. Another recent case report describes the successful treatment of a resistant PDPH to EBP with a suprazygomatic SPG block [17]. The SPG was targeted via the suprazygomatic approach, utilizing a 22G spinal needle directed toward the pterygopalatine fossa with ultrasound guidance.

We feel that the J-tip catheter approach is a noninvasive method to deliver the local anesthetic closer to the SPG ganglion to improve the block and sympathectomy. In an observational paper, Kim et al. [18] found significant changes in facial temperature when performing the SPG block with a catheter used to drip local anesthetic at the site versus the traditional topical approach with a cotton swab dipped in local anesthetic. The difference in temperature change may be indicative of the increased sympathetic block with local anesthetic applied closer than topically possible with a cotton swab.

## Conclusions

We describe the case of a pediatric patient with severe, debilitating, refractory PDPH who did not respond to conservative and medical management, multiple EBPs, or traditional nerve blocks. We recommend an SPG block with a J-tip catheter, especially for pediatric patients, because it is less invasive than the external suprazygomatic approach but more effective than the cotton swab technique and can be performed at the bedside with minimal risk in both pediatric and adult patients. In this patient with multiple epidural attempts, we believe the EBP was unable to seal the different dural puncture sites. However, the SPG block stopped the headache centrally and allowed the body time to heal on its own.
